# Special issue: Multicomponent lipid membranes—how molecular organisation leads to function

**DOI:** 10.1007/s00249-021-01535-3

**Published:** 2021-04-16

**Authors:** Bert de Groot, Andreas Janshoff, Claudia Steinem, Markus Zweckstetter

**Affiliations:** 1 Max-Planck-Institut für Biophysikalische Chemie, Am Fassberg 11, 37077 Göttingen, Germany; 2 Georg-August Universität, Institut für Physikalische Chemie, Tammannstr. 6, 37077 Göttingen, Germany; 3 Georg-August Universität, Institut für Organische und Biomolekulare Chemie, Tammannstr. 2, 37077 Göttingen, Germany; 4 Deutsches Zentrum für Neurodegenerative Erkrankungen (DZNE), Von-Siebold-Str. 3a, 37075 Göttingen, Germany; 5 Universitätsmedizin Göttingen, Abteilung Neurologie, Waldweg 33, 37073 Göttingen, Germany

From 2009 to 2020, a group of scientists, with great expertise in the area of membrane biophysics, closely worked together in the Collaborative Research Centre 803 (CRC 803, *Sonderforschungsbereich* 803), with the common goal to unravel the interactions between the large number of different lipids and specialized proteins in cellular membranes on the molecular level. The mission of the CRC 803 was to clarify how the spatial and temporal organisation of membrane components influences their function and vice versa. The findings of the CRC 803 enabled us to draw molecular pictures of how peptides and proteins in the lipid membrane as well as between two membranes lead to structures that are responsible for transport processes across membranes, and substantial topological changes comprising the fusion of two lipid bilayers. To obtain the required high spatial and temporal resolution to follow these molecular processes combined with precise control of composition, altogether, 19 projects pursued bottom-up approaches utilizing tailor-made, peptide-/protein containing model membrane systems paired with experimental and theoretical methods developments, such as high-resolution microscopy and computer simulations.

We had the great opportunity to be part of this team and decided to act as guest editors for a special issue highlighting major parts of our results. We collected 14 articles, a blend of original articles and reviews. In the first article of this special issue, the question is addressed how the chemical structure of the globoside Gb_3_ influences the phase behaviour of membranes and the binding of Shiga toxin (Sibold et al. 2020). In the second original article, the influence of different arginine derivatives on the integrity of lipid membranes is dissected by means of experimental and theoretical approaches (Verbeek et al. 2021). Three articles impressively show how EPR- and NMR-spectroscopy contribute to our understanding how simple transmembrane peptides as well as large proteins are organised in lipid membranes and interact with small molecules and membrane lipids (Tkach et al. 2021; Naibauer et al. 2021; Riviere et al. 2020). The article of de Groot and co-worker shows the potential of atomistic simulations for our understanding of transmembrane proteins and their interaction with lipids (Daday and de Groot 2020). Besides these insights into the biophysics of interactions within membranes, the CRC 803 was also able to develop new methods, such as the model-free multiscale analysis of patch-clamp recordings (Pein et al. 2021) and microscopy tools to visualize membrane interactions (Grothe et al. 2021). A large part of the CRC 803 was dedicated to elucidate the process of membrane fusion. Here, five articles contribute to our understanding of how the process of neuronal fusion takes place, identifying prominent intermediate states of fusion and quantifying the energetics of fusion (Witt et al. 2021; Mühlenbrock et al. 2020; Smirnova et al. 2021; Scheu et al.^2021^; Risselada et al. 2020). In the last article of this special issue, protein-dependent membrane remodelling is discussed (Tarasenko and Meinecke 2021).

Without the great effort of all members of the CRC 803, these fascinating results would not have been achieved. We would like to thank all participants working within the CRC 803 over the last 12 years for their invaluable input and in particular those, who participated in this special issue to finalise our endeavour. We would also like to thank the referees for their great support to ensure that this special issue fulfils high-quality standards. Many thanks also to the *European Biophysics Journal* for providing us the opportunity to present this exciting research field to a broad audience.

Bert de Groot, Andreas Janshoff, Claudia Steinem, Markus Zweckstetter.

(Guest Editors).
